# Comparison of peri- and intraoperative outcomes of open vs robotic-assisted partial nephrectomy for renal cell carcinoma: a propensity-matched analysis

**DOI:** 10.1186/s12957-023-03061-2

**Published:** 2023-06-22

**Authors:** Benedikt Hoeh, Mike Wenzel, Olivia Eckart, Felicia Fleisgarten, Cristina Cano Garcia, Jens Köllermann, Christoph Würnschimmel, Alessandro Larcher, Pierre Karakiewicz, Luis A. Kluth, Felix K. H. Chun, Philipp Mandel, Andreas Becker

**Affiliations:** 1 Department of Urology, University Hospital Frankfurt, Goethe University Frankfurt Am Main, Theodor-Stern-Kai 7, 60590 Frankfurt Am Main, Germany; 2 Young Academics in Urology (YAU) Working Group Robotic Surgery, Arnhem, The Netherlands; 3 Cancer Prognostics and Health Outcomes Unit, Division of Urology, University of Montreal Health Center, Montreal, QC Canada; 4 Dr. Senckenberg Institute of Pathology, University Hospital Frankfurt, Frankfurt Am. Main, Germany; 5 Luzerner Kantonsspital, Lucerne Hospital, Lucerne, Switzerland; 6 Division of Experimental Oncology/Unit of Urology, URI, Urological Research Institute, IRCCS San Raffaele Scientific Institute, Milan, Italy

## Abstract

**Background:**

Partial nephrectomy (PN) is the gold standard surgical treatment for resectable renal cell carcinoma (RCC) tumors. However, the decision whether a robotic (RAPN) or open PN (OPN) approach is chosen is often based on the surgeon’s individual experience and preference. To overcome the inherent selection bias when comparing peri- and postoperative outcomes of RAPN vs. OPN, a strict statistical methodology is needed.

**Materials and methods:**

We relied on an institutional tertiary-care database to identify RCC patients treated with RAPN and OPN between January 2003 and January 2021. Study endpoints were estimated blood loss (EBL), length of stay (LOS), rate of intraoperative and postoperative complications, and trifecta. In the first step of analyses, descriptive statistics and multivariable regression models (MVA) were applied. In the second step of analyses, to validate initial findings, MVA were applied after 2:1 propensity-score matching (PSM).

**Results:**

Of 615 RCC patients, 481 (78%) underwent OPN vs 134 (22%) RAPN. RAPN patients were younger and presented with a smaller tumor diameter and lower RENAL-Score sum, respectively. Median EBL was comparable, whereas LOS was shorter in RAPN vs. OPN. Both intraoperative (27 vs 6%) and Clavien-Dindo > 2 complications (11 vs 3%) were higher in OPN (both < 0.05), whereas achievement of trifecta was higher in RAPN (65 vs 54%; *p* = 0.028). In MVA, RAPN was a significant predictor for shorter LOS, lower rates of intraoperative and postoperative complications as well as higher trifecta rates. After 2:1 PSM with subsequent MVA, RAPN remained a statistical and clinical predictor for lower rates of intraoperative and postoperative complications and higher rates of trifecta achievement but not LOS.

**Conclusions:**

Differences in baseline and outcome characteristics exist between RAPN vs. OPN, probably due to selection bias. However, after applying two sets of statistical analyses, RAPN seems to be associated with more favorable outcomes regarding complications and trifecta rates.

**Supplementary Information:**

The online version contains supplementary material available at 10.1186/s12957-023-03061-2.

## Introduction

Renal cell carcinoma (RCC) European Association of Urology (EAU) guidelines recommend a nephron-sparing surgical approach with partial nephrectomy (PN) whenever surgically applicable, with the intent to reduce postoperative morbidity caused by decreased kidney function, in comparison to radical nephrectomy [1–3]. PN can be either performed with a laparoscopic, robotic-assisted (RAPN), or open (OPN) surgical approach. Today, all approaches have shown excellent oncological and trifecta outcomes (ischemia time, positive surgical margin, postoperative complications) [4–6]. In the real-world setting, the choice of approach is usually based on several factors such as the surgeon’s experience and preference, learning curve, tumor complexity, or patients’ comorbidities [7]. Therefore, especially retrospective comparisons between perioperative and postoperative complications between RAPN vs OPN may be biased by these confounders, even after multivariable analyses. Previous studies comparing RAPN vs OPN have reported lower complication rates, lower estimated blood loss, or shorter length of hospital stay (LOS) in favor of RAPN [8–12]. In fact, today, only one prospective trial is available comparing both approaches, favoring RAPN over OPN [13].

To address this void, we relied on a strict statistical methodological approach with propensity score matched (PSM) analyses and additional multivariable adjustment for remaining differences in baseline patient, tumor, and surgical characteristics. We hypothesized that RAPN may be associated with fewer peri- and postoperative complications.

## Material and methods

### Study population

After approval of the institutional review boards of the University Cancer Centre Frankfurt and the Ethical Committee at the University Hospital Frankfurt, patients treated with RAPN or OPN between January 2003 and January 2021 were retrospectively identified from our prospectively maintained RCC database. Exclusion criteria consisted of clinical suspicion of positive lymph nodes or metastases (*n* = 21), tumor suspicion regarding a transplanted kidney (*n* = 7), or bilateral tumor suspicion (*n* = 4).

### Outcome measurements

Outcomes of interest consisted of (a) estimated blood loss, (b) rate of intraoperative complication, (c) rate of postoperative complication, (d) rate of trifecta, and (e) length of stay (LOS). Medical charts were reviewed to ascertain information regarding the outlined outcomes of interest. Postoperative complications (30 days post-surgery) were classified according to the Clavien-Dindo classification and trifecta was defined as the presence of negative surgical margin and ischemia time < 25 min, as well as the absence of any postoperative complication reported [4, 14]. LOS was defined as the time from surgery to discharge from the hospital.

### Statistical analyses

The statistical analyses consisted of four steps: First, patients were tabulated according to surgical approach (RAPN vs OPN). Here, descriptive statistics included frequencies and proportions for categorical variables. Medians and interquartile ranges (IQR) were reported for continuously coded variables. The chi-square test examined the statistical significance of the differences in proportions while the Kruskal–Wallis test was used to examine differences in medians.

Further, prior to PSM, separate linear multivariable regression models were used to test for a relationship between surgical approach (RAPN vs. OPN) and (a) blood loss and (b) LOS. For estimated blood loss adjustment variables consisted of RENAL-Score (low, moderate, high), tumor diameter (per mm), intraoperative complication (yes vs no), surgeon’s volume (low, intermediate, high), intraoperative ischemia (yes vs no), year of surgery (annually), previous abdominal surgery (yes vs no), and Charlson-Comorbidity Index (CCI; continuously). For LOS, the same covariables additionally to postoperative complications (yes vs no) were used. Subsequently, separate multivariable logistic regression models were used to test for a relationship between RAPN vs OPN and (c) intraoperative complication, (d) postoperative complication, and (e) trifecta. Here, covariables consisted of RENAL-Score (as previously reported [15, 16]: low, moderate, high), tumor diameter (per mm), intraoperative complication (yes vs no), surgeon’s volume (low, intermediate, high), year of surgery (annually), previous abdominal surgery (yes vs no), and CCI (continuously). For intraoperative and postoperative complications, additional adjustment variables consisted of intraoperative ischemia (yes vs no) for both analyses and additional adjustment for intraoperative complications when postoperative complications were the outcome of interest in logistic regression models.

In the second step of our analyses, to account for underlying differences in patient and tumor compositions, a 2:1 PSM for age (per year), previous abdominal surgery (yes vs no), RENAL score sum (continuously), tumor diameter (per mm), and CCI (continuously) was performed according to previously reported methodology [17, 18]. Fourth and finally, we relied on PSM cohorts. Here, we repeated the analyses which were performed prior to PSM and described above. Adjustment remained unchanged except for the absence of previous abdominal surgery and CCI as covariables.

All tests were two-sided with a level of significance set a *p* < 0.05, and R-software environment for statistical computing and graphics (version 3.4.3) was used for all analyses.

## Results

### Descriptive characteristics of the study population

Of 615 patients treated with PN for RCC, 481 (78%) vs 134 (22%) underwent OPN vs RAPN, respectively (Table 1). RAPN patients were younger (62 vs 64 years), had more frequently undergone previous abdominal surgery (53 vs 39%), and presented with lower tumor complexity, evidenced by smaller tumor diameter (28 vs 35 mm), and lower RENAL-Score sum (7 vs 8), respectively (Table 1; all *p* < 0.05). No statistically significant differences were recorded for gender, BMI, modified CCI, or laterality.

**Table 1:** Descriptive characteristics of 615 patients treated with open (*n*=481) or robotic-assisted partial nephrectomy (*n*=134) for renal cell carcinoma at a tertiary care center from 01/2003 to 01/2021; All values are medians (IQR) or frequencies (%)

	***N***	**Overall, ** ***N*** ** = 615**	**OPN, ** ***N*** ** = 481 (78%)**	**RAPN, ** ***N*** ** = 134 (22%)**	***P*** ** value**
**Age [years]** Median (IQR)	615	63 (55, 71)	64 (55, 72)	62 (52, 70)	0.025
**Body mass index [m**^**2**^**/kg]** ***n*** (%)	565	27.4 (24.4–30.8)	27.4 (24.6–30.8)	27.1 (23.7–31.1)	0.4
**Male sex** ***n*** (%)	615	440 (72%)	339 (70%)	101 (75%)	
**Charlson Comorbidity Index**^**a**^ ***n*** (%)	614				0.13
0		330 (54%)	249 (52%)	81 (61%)	
1		122 (20%)	97 (20%)	25 (19%)	
≥ 2		162 (26%)	135 (28%)	27 (20%)	
**Tumor diameter [mm]** Median (IQR)	612	33 (24, 45)	35 (25, 49)	28 (21, 37)	< 0.001
**RENAL-score sum** Median (IQR)	432	8 (7, 9)	8 (7, 9)	7 (6, 9)	< 0.001
**RENAL-score grouped** ***n*** (%)	407				< 0.001
Low		83 (20%)	46 (15%)	37 (37%)	
Moderate		238 (58%)	184 (60%)	54 (54%)	
High		86 (21%)	77 (25%)	9 (9.0%)	
**Previous abdominal surgery** ***n*** (%)	615	259 (42%)	188 (39%)	71 (53%)	0.004
**Laterality** ***n*** (%)	615				0.2
Right		333 (54%)	253 (53%)	80 (60%)	
Left		282 (46%)	228 (47%)	54 (40%)	

*Abbreviations*: *IQR* Interquartile range, *OPN* Open partial nephrectomy, *RAPN* Robotic-assisted partial nephrectomy

^a^Modified CCI: age as co-variable excluded

### Outcomes of interest

Median estimated blood loss did not differ between RAPN vs OPN (350 vs 400 ml; *p* = 0.15), whereas LOS was shorter in RAPN compared to OPN (6 vs 7 days; *p* < 0.001; Table 2). Intraoperative complication rates were higher in OPN vs RAPN (27 vs. 6%; *p* = 0.01). For OPN, the most frequent intraoperative complications were pleural injury (*n* = 87), blood vessel injury (*n* = 25), and spleen injury (*n* = 5). For RAPN, blood vessel injury (*n* = 5) and pleural injury (*n* = 3) were the recorded intraoperative complications.

**Table 2:** Perioperative outcomes of 615 patients treated with open (*n*=481) or robotic-assisted partial nephrectomy (*n*=134) for renal cell carcinoma at a tertiary care center from 01/2003 to 01/2021; All values are medians (IQR) or frequencies (%)

	***N***	**Overall ** ***N*** ** = 615**	**OPN ** ***N*** ** = 481 (78%)**	**RAPN ** ***N*** ** = 134 (22%)**	***P*** ** value**
**Length of stay [days]** Median (IQR)	615	7 (6, 9)	7 (6, 10)	6 (5, 6)	< 0.001
**Operation time [min]** Median (IQR)	610	187 (147, 230)	186 (147, 230)	188 (144, 228)	0.7
**Blood loss [ml]** Median (IQR)	321	400 (200, 800)	400 (200, 800)	350 (200, 625)	0.15
**pT-stage** ***n*** **(%)**	591				0.020
pT1a		393 (66%)	295 (63%)	98 (80%)	
pT1b		143 (24%)	123 (26%)	20 (16%)	
pT2a		17 (2.9%)	15 (3.2%)	2 (1.6%)	
pT2b		5 (0.8%)	5 (1.1%)	0 (0%)	
≥ pT3		33 (5.6%)	30 (6.4%)	3 (2.4%)	
**Surgical margin** ***n*** (%)	588				0.3
R0		557 (95%)	444 (95%)	113 (93%)	
R1		17 (2.9%)	11 (2.4%)	6 (4.9%)	
Rx		14 (2.4%)	11 (2.4%)	3 (2.5%)	
**Surgeon’s volume** Median (IQR)	615	43 (13, 77)	35 (11, 70)	65 (40, 98)	< 0.001
**Surgeon’s volume grouped** ***n*** (%)	615				< 0.001
Low		201 (33%)	191 (40%)	10 (7.5%)	
Intermediate		90 (15%)	64 (13%)	26 (19%)	
High		324 (53%)	226 (47%)	98 (73%)	
**Intraoperative ischemia** ***n*** (%)	615				0.2
Yes		379 (62%)	290 (60%)	89 (66%)	
No		236 (38%)	191 (40%)	45 (34%)	
**Ischemia duration [min]** Median (IQR)	375	16.0 (13.0, 20.0)	16.0 (13.0, 20.0)	15.0 (12.0, 19.0)	0.2
**Transfusion intraoperative** ***n*** (%)	613	23 (3.8%)	23 (4.8%)	0 (0%)	0.010
**Transfusion postoperative** ***n*** (%)	608	50 (8.2%)	44 (9.3%)	6 (4.5%)	0.078
**Transfusion total** ***n*** (%)	608	73 (12%)	67 (14%)	6 (4.5%)	0.003
**Intraoperative complication** ***n*** (%)	615	138 (22%)	130 (27%)	8 (6.0%)	0.010
**Conversion to OPN,** ***n*** (%)	615		-	13 (9.7%)	-
**Conversion to nephrectomy** ***n*** (%)	615	55 (8.9%)	50 (10%)	5 (3.7%)	0.017
**Trifecta achievement** ***n*** (%)	615	348 (57%)	261 (54%)	87 (65%)	0.028
**Clavien-Dindo 30-days complication** ***n*** (%)	615				0.009
0		389 (63.3%)	288 (60%)	101 (75.4%)	
1–2		170 (27.6%)	141 (29.3%)	29 (21.7%)	
> 2		56 (9.1%)	52 (10.7%)	4 (2.9%)	

*Abbreviations*: *IQR* Interquartile range

Higher grade (Clavien-Dindo > 2) postoperative complication (11 vs 3%) rates were significantly higher in OPN, whereas achievement of trifecta (65 vs 54%; *p* = 0.028) was statistically significant higher in RAPN conversely (Table 2). Moreover, perioperative transfusion rates were lower in RAPN compared to OPN (4.5 vs 14%; *p* = 0.003) and fewer PN were converted into radical nephrectomy in RAPN compared to OPN (3.7 vs 10%; *p* = 0.017). No differences were recorded in the use of intraoperative ischemia (66 vs 60%; *p* = 0.2), as well as ischemia duration, if applied (median: 15 vs 16 min; *p* = 0.2).

### Multivariable linear and logistic regression models prior to PSM

Prior to PSM, RAPN did not reach significant predictor status for blood loss in multivariable linear regression analyses (Table 3). Contrary, when LOS was the outcome of interest, RAPN was an independent predictor for shorter LOS in multivariable linear regression analyses (beta: − 1.04; *p* = 0.04; Table 3). In separate multivariable logistic regression analyses, RAPN was associated with lower rates of intraoperative complications (OR [odds ratio]: 0.17; *p* < 0.001; Table 3), lower rates of postoperative complications (OR: 0.55; *p* = 0.04; Table 3) and higher rates of trifecta achievement (OR: 1.74; *p* = 0.044; Table 3).

**Table 3:** Multivariable linear regression models (estimated blood loss, LOS) and multivariable logistic regression models (intraoperative complication, postoperative complication, trifecta) in 615 patients treated with open (*n*=481) or roboticassisted partial nephrectomy (*n*=134) for renal cell carcinoma at a tertiary care center from 01/2003 to 01/2021

	**Estimated blood loss** ^a^			**LOS** ^b^			**Intraoperative complication** ^c^			**Postoperative complication** ^d^			**Trifecta** ^e^		
	**Beta**	**95% CI**	***P*** ** value**	**Beta**	**95% CI**	***P*** ** value**	**OR**	**95% CI**	***P*** ** value**	**OR**	**95% CI**	***P*** ** value**	**OR**	**95% CI**	***P*** ** value**
**Surgical approach**															
**Open**															
Robotic-assisted	11.003	− 232.647, 254.653	0.929	− 1.044	− 2.058, − 0.030	0.044	0.1736	0.0682, 0.3825	0.0001	0.5493	0.3022, 0.9761	0.0444	1.7353	1.0201, 29,949	0.0443

*Abbreviations*: *Ref* Reference, *LOS* Length of stay, *OR* Odds ratio, *95% CI* 95% confidence interval, *CCI* Charlson-Comorbidity Index

^a^Multivariable linear regression; adjusted for intraoperative complication, tumor diameter, RENAL score, intraoperative ischemia, surgeon’s volume, year, previous abdominal surgery, CCI

^b^Multivariable linear regression; adjusted for intraoperative complication, postoperative complication, tumor diameter, RENAL score, intraoperative ischemia, surgeon’s volume, year, previous abdominal surgery, CCI

^c^Multivariable logistic regression; adjusted for tumor diameter, RENAL score, intraoperative ischemia, surgeon’s volume, year, previous abdominal surgery, CCI

^d^Multivariable logistic regression; adjusted for intraoperative complication, tumor diameter, RENAL score, intraoperative ischemia, surgeon’s volume, year, previous abdominal surgery, CCI

^e^Multivariable logistic regression; adjusted for tumor diameter, RENAL score, surgeon’s volume, year of surgery, previous abdominal surgery, CCI

### PSM analyses

PSM addressed 615 patients. Of those, 204 of 481 OPN and 102 of 134 RAPN could be matched in a 1:2 fashion. No statistically significant differences according to age, BMI, CCI, tumor diameter, RENAL score, and history of previous abdominal surgery existed between OPN vs RAPN after PSM (standard mean differences: < 0.1; Supplementary Table 1).

### Multivariable linear and logistic regression models after PSM

In PSM analyses, RAPN did not reach significant predictor status for both estimated blood loss and LOS in separate multivariable linear regression models (both *p* > 0.05; Table 4). In line with the results prior to PSM, RAPN remained in PSM analyses a significant predictor for lower rates of intraoperative complications (OR: 0.16; *p* < 0.001; Table 4), lower rates of postoperative complications (OR: 0.55; *p* = 0.045; Table 4), and higher rates of trifecta achievement (OR: 1.84; *p* = 0.038; Table 4).

**Table 4:** Multivariable linear regression models (estimated blood loss, LOS) and multivariable logistic regression models (intraoperative complication, postoperative complication, trifecta) in 306 patients treated with open (*n*=204) or roboticassisted partial nephrectomy (*n*=102) for renal cell carcinoma at a tertiary care center from 01/2003 to 01/2021 after propensity score matching

	**Estimated blood loss** ^a^			**LOS** ^b^			**Intraoperative complication** ^c^			**Postoperative complication** ^d^			**Trifecta** ^e^		
	**Beta**	**95% CI**	***P*** ** value**	**Beta**	**95% CI**	***P*** ** value**	**OR**	**95% CI**	***P*** ** value**	**OR**	**95% CI**	***P*** ** value**	**OR**	**95% CI**	***P*** ** value**
**Surgical approach**															
**Open**	*Ref.*			*Ref.*			*Ref.*			*Ref.*			*Ref.*		
Robotic-assisted	30.750	− 188.789, 250.289	0.782	− 0.831	− 1.936, 0.274	0.140	0.1570	0.0605, 0.3562	< 0.001	0.5460	0.2835, 0.9431	0.0452	1.8394	1.0385, 3.3036	0.0385

Abbreviations: *Ref*, reference; *LOS*, length of stay; *OR*, odds ratio; *95% CI*, 95% confidence interval

^a^Multivariable linear regression; adjusted for intraoperative complication, tumor diameter, RENAL score, intraoperative ischemia, surgeon’s volume, year

^b^Multivariable linear regression; adjusted for intraoperative complication, postoperative complication, tumor diameter, RENAL score, intraoperative ischemia, surgeon’s volume, year

^c^Multivariable logistic regression; adjusted for tumor diameter, RENAL score, intraoperative ischemia, surgeon’s volume, year

^d^Multivariable logistic regression; adjusted for intraoperative complication, tumor diameter, RENAL score, intraoperative ischemia, surgeon’s volume, year

^e^Multivariable logistic regression; adjusted for tumor diameter, RENAL score, surgeon’s volume, year of surgery

## Discussion

In the current study, we hypothesized that RAPN may be associated with fewer peri- and postoperative complications compared to OPN. However, we also hypothesized that this observation is based on important confounders and may not be present after strict application of PSM and additional multivariable analyses adjusting for remaining baseline and surgical differences and OPN may lead to comparable results. We tested these hypotheses within our institutional RCC database and made several noteworthy observations.

First, we observed important differences in patient and tumor characteristics between RCC patients treated with RAPN vs OPN. For example, patients treated with RAPN were younger and harbored more frequently smaller tumor masses and tumor complexity measured by RENAL score. This observation clearly indicates that the decision for the surgical approach is indeed biased by patient selection and surgeons’ preferences towards less complex cases performed with RAPN. To the best of our knowledge, these observations are in agreement with all retrospective studies investigating differences between RAPN vs. OPN [19]. For example, a multicenter study on behalf of the Comité Cancer de l’Association Francaise d’Urologie by Ingels et al. also reported smaller tumor diameters < 4 cm (72 vs. 55%) and less complex tumors (44 vs. 30%), when RAPN was performed and compared to OPN [11]. Similarly, a recently published meta-analyses by Shen et al.—including 16 studies with over 3000 PN cases—also concluded the above-mentioned assumptions after data pooling [20].

Second, we observed that estimated blood loss did not statistically significantly differ when RAPN and OPN were compared. Even though there was a tendency to lower blood loss in the RAPN group compared to OPN (median blood loss: 350 vs 400 ml), results within the current study tend to be at the higher range of previously reported blood losses [12, 20, 21]. It is of note that especially estimated median blood loss for RAPN tended to be higher within the current study compared to previously reported results. For example, in a systematic review by Shen et al. comparing perioperative outcomes of RAPN and OPN, most included studies reported median blood loss lower than 200; however, some studies reported higher median estimated blood loss levels, too [20, 22]. Interestingly, however, perioperative transfusion rates were significantly lower in RAPN compared to OPN and were comparable to previously reported studies. Due to the retrospective design of the current study, differences in regards to type and exactness of blood loss measurements may be prevalent and therefore estimated blood loss tended to be higher in the current study compared to previous studies.

Moreover, LOS, as well as proportions of intraoperative complications, conversion to radical nephrectomy, and postoperative complications were significantly higher in the OPN cohort. Conversely, the trifecta rate (negative surgical margins, ischemia time < 25 min, and no postoperative complications) was higher in the RAPN cohort. In multivariable analyses, RAPN was still an independent predictor of shorter LOS, perioperative and postoperative complications and a higher chance of trifecta achievement, when adjustment for baseline patient and tumor characteristics, as well as other confounders, was performed. Comparing these results to the current literature, most previous analyses observed RAPN as a surgical approach, significantly associated with shorter LOS and shorter blood loss [20, 23]. Similarly, multiple studies and two different meta-analyses by Shen et al. and Grivas et al. on behalf of the YAU robotic working group also reported lower perioperative complication rates with RAPN, relative to OPN [8, 20, 24]. Comparing trifecta rates, we observed higher trifecta achievement in our cohort with RAPN (65 vs 54%) than with OPN and an independent predictor status after multivariable analyses in favor of RAPN. Interestingly, when trifecta achievement was dissected into its different components, differences as regards intraoperative complications (6 vs 27%, *p* = 0.01) were substantially accountable for the different rates between RAPN vs OPN. These findings are in an agreement with previously published literature. For example, Hori et al. also reported significantly higher trifecta rates in RAPN-treated patients (71 vs 51%), relative to OPN patients [10, 25].

Despite the above-made and discussed findings, multivariable analyses may not fully adjust for differences in baseline patient, tumor, and surgeons’ characteristics. For example, performance status represents an independent and strong predictor of adverse outcomes after PN so we aimed to further validate our findings with a more stringent statistical methodology [26, 27]. Therefore, in the second step of our analyses, we relied on PSM. The objective of matching was to maximally adjust for age, previous abdominal surgery, RENAL score sum, tumor diameter, and performance status with the intent of illustrating the most unbiased and the most direct effect of RAPN, relative to OPN on outcomes of interest. It is of note that propensity-score matching was performed in previous studies comparing perioperative outcomes between OPN vs RAPN. However, the type and extent of the propensity score matching differed substantially. For example, Ficarra et al. relied on propensity-score matching in order to compare perioperative outcomes in a multicenter cohort of 400 patients treated with OPN vs RAPN [21]. However, despite the approach within the current study, performance status was not included within the propensity score matching, and therefore, results might not be directly comparable between the study by Ficarra et al. and the current study [21]. Moreover, in addition to PSM, we also relied on additional multivariable adjustment for residual baseline patient, tumor, and surgeons’ differences, such as surgeons’ volume or intraoperative ischemia or year of surgery.

After applying both methodologies, we observed that LOS did no longer differ between both surgical approaches. However, and even more importantly, RAPN remained an independent predictor of lower perioperative and postoperative complications compared to OPN. Additionally, RAPN was an independent predictor for the achievement of the trifecta. These observations are noteworthy, since they reject our initial null hypotheses. Moreover, to the best of our knowledge, no previously published study has conducted two-step fashion analyses with validation of their own data in an even more stringent statistical analysis with PSM. Therefore, one can assume that the observations made in the current study can be also interpreted as an external validation of recently published large-scale studies, relying on less stringent methodology, finding also an independent predictor status for lower complications and higher trifecta rates with RAPN, relative to OPN [28, 29].

Despite the noteworthy findings, the current study is not devoid of limitations. First, despite the application of the most stringent statistical methodology for this retrospective cohort analysis, further unknown confounders may have an impact on study outcomes. Prospective studies should ideally validate our findings. Second, we relied only on non-metastatic RCC patients. However, the generalizability for cytoreductive PN or adjuvant PN in times of neoadjuvant immunotherapy is unknown [30, 31]. Third, within the current study, solely perioperative outcomes were investigated in RAPN vs OPN. It is of note that the current study did not perform cost-analyses, and therefore, cost-effectiveness conclusions regarding surgical approach (robotic-assisted vs open) cannot be drawn from the current manuscript as previously performed for urological cancer surgeries [32–34]. Fourth and finally, limitations that are inherent due to the retrospective nature of the study might be present, and thus, results must be interpreted accordingly.

## Conclusion

Taken together, when comparing RAPN vs OPN in a high-volume center within a period of almost two decades, important differences in patient and tumor characteristics towards less complex RCC surgical procedures in RAPN were observed. However, even after PSM and additional multivariable adjustment to balance confounders and report in the most unbiased fashion, RAPN was associated with a significantly lower probability of perioperative and postoperative complications, as well as an independently higher chance of trifecta achievement. These observations may be used for patients’ counseling and surgical procedure planning purposes.

## Supplementary Information

**Additional file 1: Supplementary Table 1.** Descriptive characteristics of 306 patients treated with open (*n*=204) or robotic-assisted partial nephrectomy (*n*=102) for renal cell carcinoma at a tertiary care center from 01/2003 to 01/2021 following propensity score matching (ratio 2:1); All values are medians (IQR) or frequencies (%).

**Additional file 2: Supplementary Table 2.** Perioperative outcomes of 306 patients treated with open (*n*=204) or robotic-assisted partial nephrectomy (*n*=102) for renal cell carcinoma at a tertiary care center from 01/2003 to 01/2021 after propensity score matching (ratio 2:1); All values are medians (IQR) or frequencies (%).

## Data Availability

All datasets generated for this study are included in the manuscript.
