# Supplementary material for: Comparison of peri- and intraoperative outcomes of open vs robotic-assisted partial nephrectomy for renal cell carcinoma: a propensity-matched analysis
Source: World J Surg Oncol. 2023 Jun 22;21:189. doi: 10.1186/s12957-023-03061-2 (PMC10286329; doi:10.1186/s12957-023-03061-2)
Supplement: Supplementary file 1 — Additional file 1: Supplementary Table 1. Descriptive characteristics of 306 patients treated with open (n=204) or robotic-assisted partial nephrectomy (n=102) for renal cell carcinoma at a tertiary care center from 01/2003 to 01/2021 following propensity score matching (ratio 2:1); All values are medians (IQR) or frequencies (%). [file 12957_2023_3061_MOESM1_ESM.docx]

|  |  |  |  |  |
| --- | --- | --- | --- | --- |
|  | **N** | **Overall,**  N = 306 | **OPN,**  N = 204 (67%) | **RAPN,**  N = 102 (33%) |
| Age [years]  Median (IQR) | 306 | 64 (55, 71) | 64 (55, 71) | 63 (54, 71) |
| male sex  n (%) | 306 | 227 (74%) | 148 (73%) | 79 (77%) |
| Body Mass Index [m^2^/kg]  Median (IQR) | 302 | 26.9 (24.2, 30,4) | 27.1 (24.5, 30.5) | 26.9 (23.5, 30.3) |
| Charlson Comorbidity Index*  n (%) | 306 |  |  |  |
| 0 |  | 170 (56%) | 110 (54%) | 60 (59%) |
| 1 |  | 59 (19%) | 39 (19%) | 20 (20%) |
| ≥2 |  | 77 (25%) | 55 (27%) | 22 (22%) |
| Tumor diameter [mm]  Median (IQR) | 306 | 30 (21, 39) | 30 (21, 39) | 28 (21, 38) |
| RENAL-Score sum  Median (IQR) | 306 | 8 (6, 9) | 8 (6, 9) | 7 (6, 9) |
| RENAL-Score grouped  n (%) | 286 |  |  |  |
| Low |  | 78 (27%) | 42 (22%) | 36 (36%) |
| Moderate |  | 179 (63%) | 125 (67%) | 54 (55%) |
| High |  | 29 (10%) | 20 (11%) | 9 (9.1%) |
| History of previous abdominal surgery  n (%) | 306 | 164 (54%) | 108 (53%) | 56 (55%) |
| Laterality  n (%) | 306 |  |  |  |
| Right |  | 161 (53%) | 106 (52%) | 55 (54%) |
| Left |  | 145 (47%) | 98 (48%) | 47 (46%) |

Abbreviations:

IQR=Interquartile range; OPN= open partial nephrectomy; RAPN= robotic-assisted partial nephrectomy;

* Modified CCI: Age as co-variable excluded
