# Supplementary material for: Comparison of peri- and intraoperative outcomes of open vs robotic-assisted partial nephrectomy for renal cell carcinoma: a propensity-matched analysis
Source: World J Surg Oncol. 2023 Jun 22;21:189. doi: 10.1186/s12957-023-03061-2 (PMC10286329; doi:10.1186/s12957-023-03061-2)
Supplement: Supplementary file 2 — Additional file 2: Supplementary Table 2. Perioperative outcomes of 306 patients treated with open (n=204) or robotic-assisted partial nephrectomy (n=102) for renal cell carcinoma at a tertiary care center from 01/2003 to 01/2021 after propensity score matching (ratio 2:1); All values are medians (IQR) or frequencies (%). [file 12957_2023_3061_MOESM2_ESM.docx]

|  |  |  |  |  |  |
| --- | --- | --- | --- | --- | --- |
|  | **N** | **Overall,**  N = 306 | **OPN,**  N = 204 (67%) | **RAPN,**  N = 102 (33%) | **p-value** |
| Length of stay [days]  Median (IQR) | 306 | 6 (5, 8) | 7 (6, 8) | 6 (5, 6) | <0.001 |
| Operation time [min]  Median (IQR) | 304 | 191 (150, 231) | 185 (149, 227) | 205 (153, 245) | 0.094 |
| Blood loss [ml]  Median (IQR) | 163 | 400 (200, 700) | 400 (200, 700) | 400 (200, 650) | 0.9 |
| pT-stage  n (%) | 299 |  |  |  | 0.6 |
| pT1a |  | 235 (79%) | 158 (79%) | 77 (78%) |  |
| pT1b |  | 44 (15%) | 27 (14%) | 17 (17%) |  |
| pT2a |  | 4 (1.3%) | 2 (1.0%) | 2 (2.0%) |  |
| pT2b |  | 2 (0.7%) | 2 (1.0%) | 0 (0%) |  |
| ≥pT3 |  | 14 (4.7%) | 11 (5.5%) | 3 (3.0%) |  |
| Surgical margin  n (%) | 297 |  |  |  | >0.9 |
| R0 |  | 277 (93%) | 185 (93%) | 92 (94%) |  |
| R1 |  | 12 (4.0%) | 8 (4.0%) | 4 (4.1%) |  |
| Rx |  | 8 (2.7%) | 6 (3.0%) | 2 (2.0%) |  |
| Surgeon’s volume  Median (IQR) | 306 | 45 (16, 78) | 34 (11, 66) | 66 (39, 96) | <0.001 |
| Surgeon’s volume  n (%) | 306 |  |  |  | <0.001 |
| Low |  | 88 (29%) | 81 (40%) | 7 (6.9%) |  |
| Intermediate |  | 49 (16%) | 28 (14%) | 21 (21%) |  |
| High |  | 169 (55%) | 95 (47%) | 74 (73%) |  |
| Intraoperative ischemia  n (%) | 306 |  |  |  | 0.2 |
| Yes |  | 191 (62%) | 122 (60%) | 69 (68%) |  |
| No |  | 115 (38%) | 82 (40%) | 33 (32%) |  |
| Ischemia duration [min]  Median (IQR) | 190 | 15.0 (12.0, 18.0) | 15.0 (12.0, 18.0) | 15.0 (12.0, 20.0) | 0.6 |
| Transfusion intraoperative  n (%) | 305 | 4 (1.3%) | 4 (2.0%) | 0 (0%) | 0.3 |
| Transfusion postoperative  n (%) | 304 | 18 (5.9%) | 12 (5.9%) | 6 (5.9%) | >0.9 |
| Transfusion total  n (%) | 304 | 22 (7.2%) | 16 (7.9%) | 6 (5.9%) | 0.5 |
| Intraoperative complication  n (%) | 306 | 66 (22%) | 59 (29%) | 7 (6.9%) | <0.001 |
| Conversion to OPN,  n (%) | 306 | 11 (3.6%) | 0 (0%) | 11 (11%) | - |
| Conversion to nephrectomy  n (%) | 306 | 10 (3.3%) | 6 (2.9%) | 4 (3.9%) | 0.7 |
| Trifecta achievement  n (%) | 306 | 188 (61%) | 116 (57%) | 72 (71%) | 0.020 |
| Clavien-Dindo 30-days complication  n (%) | 306 |  |  |  | 0.013 |
| 0 |  | 208 (68%) | 130 (64%) | 78 (76.5%) |  |
| 1-2 |  | 71 (23.2%) | 49 (23.9%) | 22 (21.6%) |  |
| >2 |  | 27 (8.8%) | 25 (12.1%) | 2 (1.9%) |  |

**Abbreviations:**

IQR=interquartile range
